# The European Rare Kidney Disease Registry (ERKReg): objectives, design and initial results

**DOI:** 10.1186/s13023-021-01872-8

**Published:** 2021-06-02

**Authors:** Giulia Bassanese, Tanja Wlodkowski, Aude Servais, Laurence Heidet, Dario Roccatello, Francesco Emma, Elena Levtchenko, Gema Ariceta, Justine Bacchetta, Giovambattista Capasso, Augustina Jankauskiene, Marius Miglinas, Pietro Manuel Ferraro, Giovanni Montini, Jun Oh, Stephane Decramer, Tanja Kersnik Levart, Jack Wetzels, Elisabeth Cornelissen, Olivier Devuyst, Aleksandra Zurowska, Lars Pape, Anja Buescher, Dieter Haffner, Natasa Marcun Varda, Gian Marco Ghiggeri, Giuseppe Remuzzi, Martin Konrad, Germana Longo, Detlef Bockenhauer, Atif Awan, Ilze Andersone, Jaap W. Groothoff, Franz Schaefer

**Affiliations:** 1 Division of Pediatric Nephrology, Center for Pediatrics and Adolescent Medicine, University of Heidelberg, Heidelberg, Germany; 2 Nephrology and Transplantation Department, Centre de Référence des Maladies Rénales Héréditaires de l’Enfant et de l’Adulte, Necker University Hospital, APHP, Université de Paris, Paris, France; 3 APHP, Pediatric Nephrology Unit, Centre de Référence des Maladies Rénales Héréditaires de l’Enfant et de l’Adulte (MARHEA), Hôpital Universitaire Necker-Enfants Malades, 75015 Paris, France; 4 Nephrology and Dialysis Unit, San Giovanni Hub Hospital and Department of Clinical and Biological Sciences, University of Turin, Turin, Italy; 5 Division of Nephrology, Bambino Gesù Children’s Hospital IRCCS, Rome, Italy; 6 Department of Pediatric Nephrology and Development and Regeneration, University Hospitals Leuven,, University of Leuven, Leuven, Belgium; 7 Department of Paediatric Nephrology, Hospital Universitario Vall d’Hebron, Barcelona, Spain; 8 Department of Paediatric Nephrology, Rheumatology and Dermatology, Reference Center for Rare Renal Diseases, Reference Center for Rare Diseases of Calcium and Phosphorus, University Children’s Hospital, Lyon, France; 9 Department of Translational Medical Sciences, University Luigi Vanvitelli, Naples, Italy; 10 Vilnius University Hospital Santaros Klinikos, Pediatric Center, Vilnius University, Vilnius, Lithuania; 11 Vilnius University Hospital Santaros Klinikos, Nephrology Center, Vilnius University, Vilnius, Lithuania; 12 U.O.S. Terapia Conservativa della Malattia Renale Cronica, U.O.C. Nefrologia, Dipartimento di Scienze Mediche e Chirurgiche, Fondazione Policlinico Universitario A. Gemelli IRCCS, Rome, Italy; 13 Pediatric Nephrology, Dialysis and Transplant Unit, Fondazione Ca’ Granda IRCCS, Policlinico di Milano, Milan, Italy; 14 Department of Clinical Sciences and Community Health, University of Milano, Milan, Italy; 15 Department of Pediatrics, University Medical Center Hamburg-Eppendorf, Hamburg, Germany; 16 Pediatric Nephrology, Internal Medicine and Rhumatology, Southwest Renal Rares Diseases Centre (SORARE), University Children’s Hospital, Toulouse, France; 17 Pediatric Nephrology Department, Children’s Hospital, University Medical Centre Ljubljana, Ljubljana, Slovenia; 18 Radboud University Medical Center, Nijmegen, Netherlands; 19 Department of Pediatric Nephrology, Radboudumc, Amalia Children’s Hospital, Nijmegen, Netherlands; 20 Division of Nephrology, UCLouvain Medical School, Brussels, Belgium; 21 Mechanisms of Inherited Kidney Disorders Group, Institute of Physiology, University of Zurich, Zurich, Switzerland; 22 Department of Pediatrics, Nephrology and Hypertension, Medical University of Gdansk, Gdańsk, Poland; 23 Department of Pediatrics II, University Hospital of Essen, University Duisburg-Essen, Essen, Germany; 24 Department of Pediatric Kidney, Liver and Metabolic Diseases, Hannover Medical School, Hannover, Germany; 25 Department of Pediatrics, University Medical Center Maribor, Maribor, Slovenia; 26 Division of Nephrology, Dialysis and Transplantation, Scientific Institute for Research and Health Care, IRCCS Istituto Giannina Gaslini, Genoa, Italy; 27 Clinical Research Centre for Rare Diseases ‘Aldo e Cele Daccò’, Istituto di Ricerche Farmacologiche Mario Negri IRCCS, Bergamo, Italy; 28 Department of Paediatric Nephrology, University Children’s Hospital, Muenster, Germany; 29 Pediatric Nephrology, Dialysis and Transplant Unit, Department of Women’s and Children’s Health, University Hospital of Padua, Padua, Italy; 30 Department of Renal Medicine, University College London and Paediatric Nephrology Unit, Great Ormond Street Hospital for Children, NHS Foundation Trust, London, UK; 31 Department of Nephrology, Children’s Health Ireland, Dublin, Ireland; 32 Pediatric Clinic, Children’s Clinical University Hospital, Riga, Latvia; 33 Department of Pediatric Nephrology, Amsterdam University Medical Center, Amsterdam, The Netherlands

**Keywords:** European Rare Kidney Disease Reference Network (ERKNet), Registry, Epidemiology, Nephrology, Pediatric nephrology

## Abstract

**Background:**

The European Rare Kidney Disease Reference Network (ERKNet) recently established ERKReg, a Web-based registry for all patients with rare kidney diseases. The main objectives of this core registry are to generate epidemiological information, identify current patient cohort for clinical research, explore diagnostic and therapeutic management practices, and monitor treatment performance and patient’s outcomes. The registry has a modular design that allows to integrate comprehensive disease-specific registries as extensions to the core database. The diagnosis (Orphacode) and diagnostic information (clinical, imaging, histopathological, biochemical, immunological and genetic) are recorded. Anthropometric, kidney function, and disease-specific management and outcome items informing a set of 61 key performance indicators (KPIs) are obtained annually. Data quality is ensured by automated plausibility checks upon data entry and regular offline database checks prompting queries. Centre KPI statistics and benchmarking are calculated automatically.

**Results:**

Within the first 24 months since its launch, 7607 patients were enrolled to the registry at 45 pediatric and 12 specialized adult nephrology units from 21 countries. A kidney disease diagnosis had been established in 97.1% of these patients at time of enrolment. While 199 individual disease entities were reported by Orphacode, 50% of the cohort could be classified with 11, 80% with 43 and 95% with 92 codes. Two kidney diagnoses were assigned in 6.5% of patients; 5.9% suffered from syndromic disease. Whereas glomerulopathies (54.8%) and ciliopathies including autosomal dominant polycystic kidney disease (ADPKD) (31.5%) were the predominant disease groups among adults, the pediatric disease spectrum encompassed congenital anomalies of the kidney and urinary tract (CAKUT) (33.7%), glomerulopathies (30.7%), ciliopathies (14.0%), tubulopathies (9.2%), thrombotic microangiopathies (5.6%), and metabolic nephropathies (4.1%). Genetically confirmed diagnoses were reported in 24% of all pediatric and 12% adult patients, whereas glomerulopathies had been confirmed by kidney biopsy in 80.4% adult versus 38.5% pediatric glomerulopathy cases.

**Conclusions:**

ERKReg is a rapidly growing source of epidemiological information and patient cohorts for clinical research, and an innovative tool to monitor management quality and patient outcomes.

**Supplementary Information:**

The online version contains supplementary material available at 10.1186/s13023-021-01872-8.

## Background

Rare kidney diseases comprise more than 300 inherited, congenital or acquired disorders, which altogether affect at least two million European citizens [1]. More than ten percent of adults and nearly all children with end stage kidney disease (ESKD) suffer from a rare kidney disease [2]. The low disease incidence puts patients at risk of late, missed or erroneous diagnosis and delayed referral to expert centres and limits even the expertise of specialists, leading to suboptimal therapeutic management and compromised long-term outcomes.

The European Rare Kidney Disease Reference Network (ERKNet) is one of 24 European Reference Networks (ERNs) created by the European Union in 2017 [3]. ERKNet is a consortium of currently 32 pediatric and 20 adult nephrology centres with specific expertise in rare kidney diseases. ERKNet aims to improve the quality of patient management by systematic education of healthcare professionals, the development and implementation of best practice guidelines, virtual expert consultations for challenging cases, and promotion of clinical research activities [4]. In order to document the clinical impact of their efforts, ERKNet members have committed to continuously monitor key performance and outcome indicators (KPIs) in their patients.

Patient registries are a crucial component of patient management and clinical research in the rare disease area [5, 6]. An internal survey performed in 2017 revealed that the ERKNet centres were active in more than 60 mostly disease-specific registries with mostly regional or national patient coverage or voluntary participation. None of the registries was used in all ERKNet centres and few provided monitoring of relevant disease or treatment specific performance and outcome measures. In order to counteract this fragmentation and improve the quantity and quality of captured information, the Network decided to create the European Rare Kidney Disease Registry (ERKReg). Here, we provide a detailed report of the registry design and implementation and characterise the patient population assembled during the registry’s first 2 years of operation.

### Registry scope and objectives

ERKReg aims to collect disease and treatment related information from all patients with rare kidney diseases who are followed at expert centres. Core demographic and diagnostic information as well as selected disease- and treatment-specific prospective follow-up data are collected. The main objectives of the registry encompass the provision of demographic disease information, the assessment of natural and therapeutically modified disease outcomes, the identification of patient cohorts suitable for diagnostic and interventional research, and the longitudinal monitoring of participating centres regarding disease-specific key performance and outcome indicators. Moreover, the registry serves as a platform for sub-registries (registry studies) that will collect more detailed information on individual rare kidney diseases or disease groups.

### Registry design and protocol

**Fig. 1:**
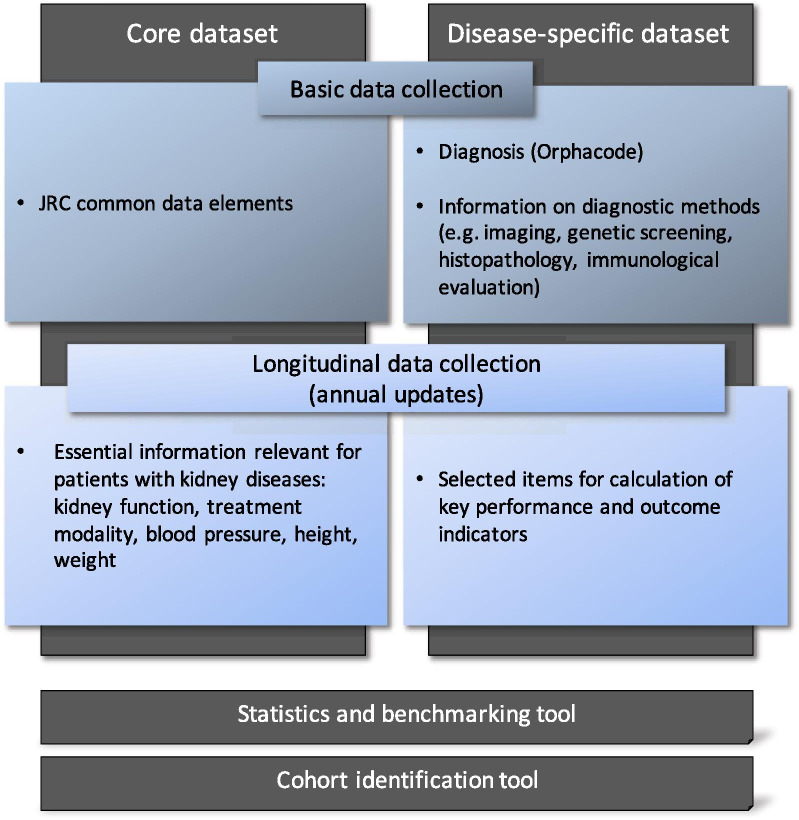
Registry design and structure

The contributing centres are requested to prospectively enrol all, both prevalent and incident, patients seen for the diagnostic and therapeutic management of a rare kidney disease in the registry. The registry contains a basic and a follow-up module (Fig. 1). The basic section captures demographic and diagnostic information, as well as a ‘termination’ section where the date and causes of follow-up discontinuation can be entered. Patients with a suspected but as yet undiagnosed rare kidney disease can be provisionally entered in an abbreviated case report form, which can be transformed to a full entry once the diagnosis has been established. The follow-up module captures general information regarding kidney function, associated comorbidities and treatment related information. In addition, selected disease-specific information is queried that allows to assess key performance and outcome indicators. The longitudinal module is updated annually.

### Registry population

All patients with a primary rare disease of the kidney or a syndromic disease associated with kidney manifestations are eligible for inclusion in the registry, irrespective of current age, age at disease presentation, disease duration, current kidney function and treatment status (conservative, dialysis, kidney transplantation). The main target disease groups include primary inherited and immune-mediated glomerulopathies and tubulopathies, systemic disorders (metabolic diseases, vasculitis) affecting glomerular and/or tubular function, ciliopathies with a renal phenotype (including autosomal dominant polycystic kidney disease and autosomal recessive polycystic kidney disease), congenital anomalies of the kidney and urinary tract (CAKUT), thrombotic microangiopathies affecting the kidneys, and rare causes of arterial hypertension.

Participation in the ERKReg registry is a core commitment of all member and Affiliated Partner centres of ERKNet. Completeness of recruitment is monitored by comparison of the cumulative enrolment figures with the numbers of total and incident patient figures provided by the centres at time of their application for membership or affiliation to ERKNet. In addition to the ERKNet member and Affiliated partner sites, participation in ERKReg (and any sub-registries as described below) is also open to interested centres outside ERKNet.

### Integration of rare kidney disease registry studies (sub-registries)

The database has a modular structure that allows to integrate disease-specific sub-registries in which more detailed phenotype and prospective treatment and outcome information is captured. The first sub-registry integrated in ERKReg is the distal renal tubular acidosis (dRTA) registry promoted by the European Society of Pedatric Nephrology (ESPN). When a patient with dRTA is enrolled in ERKReg, an optional sub-registry extension module with 29 additional data fields can be activated by the user. Further sub-registries for individual diseases (cystinuria, pediatric SLE nephritis) are currently under construction.

### Data elements

The Registry is comprised of two datasets: A **core dataset** is to be provided for all patients in the registry; it includes the Common Data Elements (CDE) defined by the Joint Research Centre (JRC) (Additional file 1: Table S1), as well as essential information relevant to all patients with rare kidney diseases such as the current kidney function (estimated glomerular filtration rate, eGFR) and treatment modality (conservative, dialysis, transplant) [7]. The Orphanet nomenclature is used to classify clinical diagnoses, and the Human Genome Variation Society (HGVS) terminology for the classification of gene variants.

In addition **disease-specific data** is captured, which comprises the diagnostic information on which the diagnosis is based, such as the genetic, histopathological, biochemical and imaging features including the date when this information was obtained. Moreover, selected disease- or disease group-specific items are collected that allow monitoring a set of 61 key performance and outcome indicators (KPIs) (Additional file 2: Table S2). The KPIs were selected by the ERKNet expert workgroups in an iterative Delphi consensus process, based on an analysis of the clinical practice guidelines endorsed by ERKNet.

The complete data dictionary of the Registry is available online (http://erkreg-metadata.erknet.org).

### Data collection and data quality management

Data is collected and entered by trained medical staff at each contributing site into online case report forms accessible through the registry website (www.registry.erknet.org). Conditional data entry menus are displayed based on the clinical diagnosis and patient age (pediatric or adult).

The users are trained in the use of the system by the registry coordination team in virtual workshops and regular ‘registry user days’. A user’s manual and FAQ document are also available through the website, and user queries are answered by a registry helpdesk.

Automated data entry checks using pre-defined plausibility ranges prevent saving of biologically or technically implausible data. As a second layer of quality control, more refined data consistency evaluations are performed and queries sent at regular intervals (e.g. implausible results for KPIs).

### Data reporting and access

The general characteristics of the registry cohort, including the distribution of the most common diagnoses, are published in Annual Reports posted on the registry website.

The local registry investigators can view continuously updated Key Performance Indicator statistics for the patients of their centre in comparison with the total registry patient cohort on the website (KPI benchmarking). In addition to the online reporting module, all investigators receive semi-annual reports of their centre’s current KPI statistics.

The registry investigators have continuous access to their own centre’s data, which can be downloaded using the export function of the platform. In addition, registry investigators of sites that enrolled at least 50 patients to the registry have access to a ‘Cohort Finder’ tool which allows to count patients by diagnosis or affected gene with an option to filter by current patient age, CKD (chronic kidney disease) stage and treatment modality.

Further data access by registry investigators and third parties is subject to review and approval by the Registry Board (see below). Data access requests can be submitted using a request form. Only aggregated results or fully anonymized patient-level data are transmitted for research purposes.

### Governance

The registry is governed by the Registry Board composed of the ERKNet Coordinator, four expert members from different thematic working groups of ERKNet, at least one representative of the sub-registries, the chair of the ERKNet Patient Advocacy Group (ePAG), and the chairs of the ERA-EDTA Working Group for Inherited Kidney Diseases (WGIKD) and the ESPN Working Group for Inherited Renal Disorders. The Registry Board is responsible for organizing and supervising the conduct of the registry. It defines the annual report contents and decides about internal and external data analysis requests. The Registry Board also serves as the primary contact for interactions with sponsors.

### Data privacy and protection

All patient data are entered and stored in a pseudonymised fashion. Only the authorized local user is able to identify the patient through an identification code, automatically generated at the time of patient registration. Access of authorized users to the registry is controlled by assignment of a secure, individualized password. A hierarchical access authorization system is implemented with super-administrator, administrator and centre user levels.

The Registry is fully compliant with the Note for Guidance on Good Clinical Practices (CPMP/ICH/135/95) [8], the EU General Data Protection Regulation (GDPR) [9] and any national rules and regulations regarding data protection. The informed consent form, based on a generic template provided by the EU for use by the ERNs, was approved by the local institutional review boards as required by local and national regulations.

Data sharing agreements defining the terms and conditions of participation in the Registry were signed by Heidelberg University Hospital, the registry lead institution, and each contributing centre.

### Technical implementation

The registry website was programmed in HTML/PHP, CSS and JavaScript. The data is stored in a SQL database hosted on a commercial server (IONOS 1&1, Montabaur, Germany), inaccessible to non-authorized personnel or entities. Regular back-ups of the database are made.

## Results

### Registry participation

**Fig. 2:**
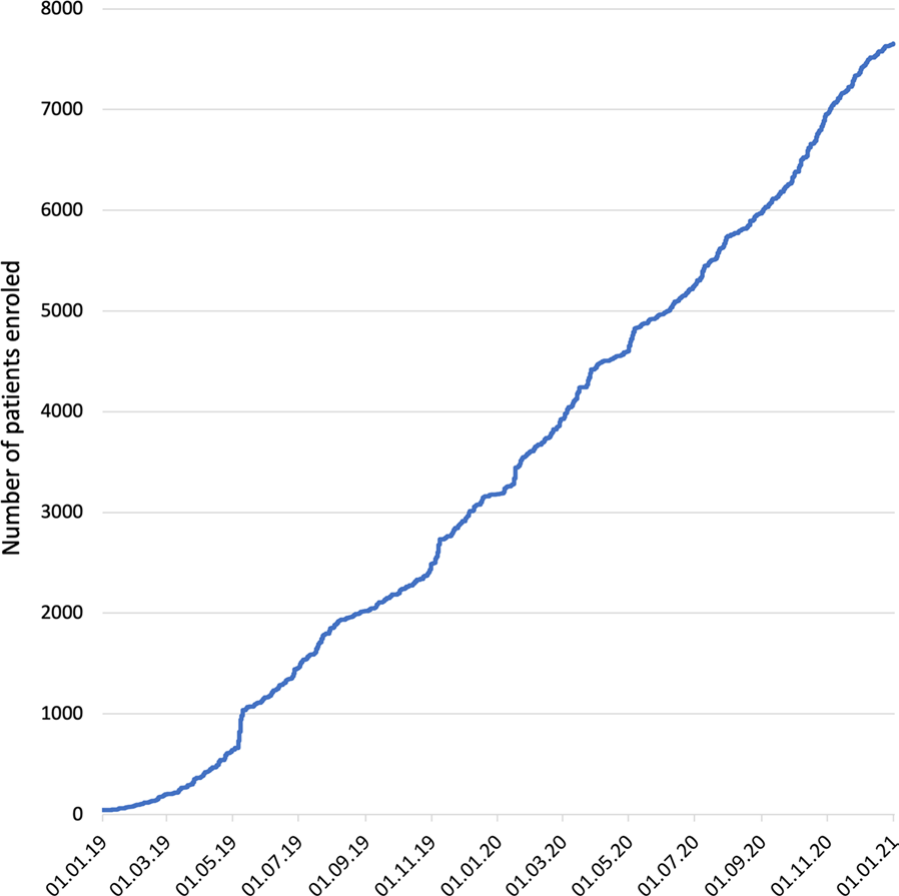
Cumulative patient enrolment in ERKReg registry

After the launch of the registry in January 2019, both ERKNet and external centres gradually started enrolling patients (Fig. 2). The number of active healthcare providers (HCPs) was 25, 30, 36, and 49 at the end of the second and fourth quarters of 2019 and 2020, respectively. The participating HCPs comprise 27 ERKNet members (contributing 92.5% of patients), 6 ERKNet Affiliated Partners (5.2% of patients), and 16 external centres (2.3% of patients).

**Fig. 3:**
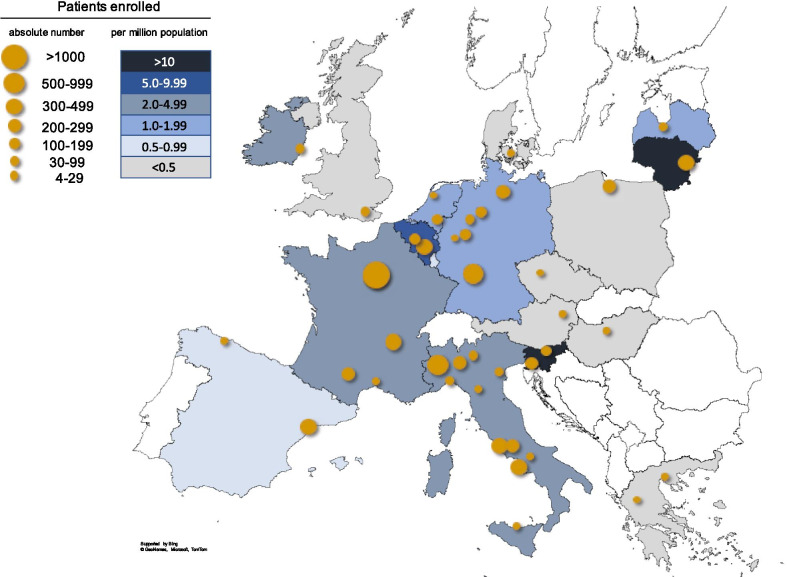
Centers and European countries contributing to the registry. Symbol size reflects number of patients enrolled per center. Country colours indicate number of patients enrolled per million population (see legend). Patient contribution from non-European countries (4 countries, 88 patients) is not included in the graph

The centres are located in 21 countries, including Italy (n = 11), Germany (n = 6), Turkey (n = 6), France (n = 4), the Netherlands (n = 2), Belgium (n = 2), Greece (n = 2), Spain (n = 2), Slovenia (n = 2), Austria, Czechia, Denmark, Hungary, Iran, Ireland, Latvia, Lithuania, Poland, Russia, the UK, and the UAE (n = 1 each). While the largest contributing countries in terms of absolute patient numbers were Italy (n = 2340), France (n = 1938) and Germany (n = 1143), the highest number of patients relative to population size was enrolled in Slovenia (16.2 per million population, pmp) and Lithuania (10.7 pmp) (Fig. 3).

Contributions are received from 45 pediatric and 12 adult nephrology units, with both pediatric and adult nephrology units being active at 7 HCPs. Data entries have been made by 101 individual users to date, with a single user being active in 27 centres, two users in eight centres, three users in four centres, four users in four centres, five users in three centres, seven users in one and eight users in one centre.

### Renal diagnoses

The Orphacode classification system was adopted for the assignment of diagnoses. A total of 335 Orphacodes designating kidney diseases or systemic disorders with a renal phenotype were identified from the thesaurus [10]. In addition, 21 entities currently not represented in the Orphacode system were included in the selection; these included several “not otherwise specified” and “by other cause” sub-categories which are not supported by OrphaNet. The complete rare kidney disease catalogue can be downloaded from the ERKReg website [11].

**Fig. 4:**
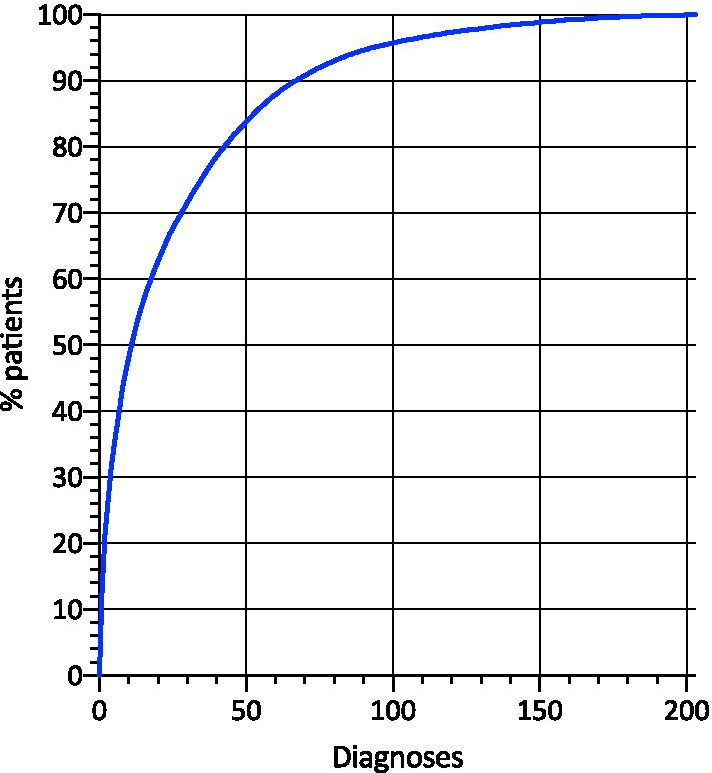
Cumulative fraction of patients identified with a given number of diagnoses ranked by frequency

Out of 7607 patients enrolled to date, 7385 (97.1%) have an established kidney disease diagnosis. Of the 335 renal disease codes, 199 were utilized to code these patients. As shown in Fig. 4, 50% of the patient population could be classified with 11 codes, 80% with 43 codes and 95% with 92 codes. The 92 most common renal disease codes are listed in Table 1.

**Table 1:** The 92 most common individual diagnoses, covering 95% of the registry cohort. Codes marked with an asterisk are internal codes for entities currently not represented in OrphaNet

Orphacode	Diagnosis	N	%	Pediatric	Adult
CAKUT (OC 93545)		1781	23.4	1719	62
93110	Posterior urethral valve	258	3.3	254	4
93173	Renal dysplasia, bilateral	227	2.9	224	3
9999969*	Vesicoureteric reflux, high-grade	216	2.8	197	19
2190	Congenital hydronephrosis	159	2.1	158	1
93100	Renal agenesis, unilateral	130	1.7	124	6
97363	Unilateral multicystic dysplastic kidney	121	1.6	121	
97362	Renal hypoplasia, bilateral	103	1.4	98	5
93172	Renal dysplasia, unilateral	84	1.1	85	
97361	Renal hypoplasia, unilateral	76	1.0	73	2
97364	Bilateral multicystic dysplastic kidney	60	0.8	59	1
9999968*	Neurogenic bladder, congenital or acquired	58	0.8	57	1
289365	Vesicoureteric reflux, familial	36	0.5	30	6
93111	RCAD (Renal cysts and diabetes) syndrome	34	0.4	33	1
9999985*	Ureteropelvic junction obstruction (bilateral or in solitary kidney)	30	0.4	29	1
887	VACTERL/VATER association	19	0.2	19	
238646	Congenital primary megaureter, obstructed form	19	0.2	19	
238650	Congenital primary megaureter, refluxing form	18	0.2	18	
238654	Congenital primary megaureter, nonrefluxing and unobstructed form	14	0.2	14	
2970	Prune belly syndrome	14	0.2	14	
238637	Megacystis-megaureter syndrome	10	0.1	9	1
567	Di George syndrome (22q11.2 deletion)	10	0.1	10	
107	BOR (branchio-oto-renal) syndrome	7	0.09	6	1
9999986*	Congenital primary megaureter, refluxing and obstructed	7	0.09	6	1
2237	HDR (Hypoparathyroidism-deafness-renal disease) syndrome	7	0.09		
Ciliopathies (OC 93587)		1406	18.4	684	722
730	Autosomal dominant polycystic kidney disease	945	12.4	312	633
731	Autosomal recessive polycystic kidney disease	150	2.0	146	4
805	Tuberous sclerosis complex	67	0.9	40	27
110	Bardet–Biedl syndrome	58	0.8	22	36
93592	Juvenile nephronophthisis	56	0.7	55	1
93591	Infantile nephronophthisis	32	0.4	31	1
93111	RCAD (Renal cysts and diabetes) syndrome	24	0.3	18	6
2318	Joubert syndrome with oculorenal defect	15	0.2	15	
Glomerulopathies (OC 93548)		2816	37.0	1561	1255
69061	Idiopathic steroid-sensitive nephrotic syndrome	643	8.5	540	130
9999982*	IgA nephropathy	368	4.8	103	265
97560	Membranous nephropathy	346	4.6	25	321
88917	Alport syndrome, X-linked	219	2.9	159	60
567546	Idiopathic steroid-sensitive nephrotic syndrome with secondary steroid resistance	95	1.2	48	47
656	Genetic steroid-resistant nephrotic syndrome	92	1.2	86	6
567552	Idiopathic steroid-resistant nephrotic syndrome with sensitivity to second-line immunouppressive therapy	90	1.2	76	14
536	SLE nephritis	78	1.0	11	67
329931	C3 glomerulonephritis	75	1.0	58	17
761	Immunoglobulin A vasculitis (Henoch Schönlein nephritis)	67	0.9	60	7
567550	Idiopathic multidrug-resistant nephrotic syndrome	56	0.7	35	21
93552	Pediatric systemic lupus erythematosus	49	0.7	49	
567544	Idiopathic non-lupus full-house nephropathy	49	0.7	15	34
9999975*	Immune complex associated membranoproliferative glomerulonephritis, not otherwise specified	48	0.6	23	25
97563	Pauci-immune glomerulonephritis with ANCA	44	0.6	16	28
88918	Alport syndrome, autosomal dominant	43	0.6	16	27
97556	Congenital nephrotic syndrome, no genetic cause specified	37	0.5	35	2
839	Congenital nephrotic syndrome, Finnish type	36	0.5	36	
88919	Alport syndrome, autosomal recessive	35	0.5	23	12
900	Granulomatosis with polyangiitis	34	0.5	6	28
329903	Immunoglobulin-mediated membranoproliferative glomerulonephritis	30	0.4	8	22
220	Denys–Drash syndrome	29	0.4	29	
727	Microscopic polyangiitis	27	0.3	3	24
9999976*	Microscopic (including familial) hematuria	23	0.3	16	7
97564	Pauci-immune glomerulonephritis without ANCA	17	0.3	5	12
91138	Cryoglobulinemic vasculitis	17	0.2		17
85445	AA amyloidosis	14	0.2		14
93571	Dense deposit disease	14	0.2	11	3
9999972*	Nephrotic syndrome, syndromic, not otherwise specified	13	0.2	13	
9999977*	Collagenopathy, not further specified	13	0.2	1	12
85443	AL amyloidosis	11	0.1		11
Tubulopathies (OC 93603)		681	8.9	539	141
402041	Autosomal recessive distal renal tubular acidosis	139	1.8	133	6
358	Gitelman syndrome	82	1.0	52	30
214	Cystinuria	81	1.0	31	50
112	Bartter syndrome	76	0.9	64	12
2197	Hypercalciuria, idiopathic	53	0.7	43	10
89936	X-linked hypophosphatemia	45	0.6	45	
223	Nephrogenic diabetes insipidus	43	0.6	36	7
93608	Autosomal dominant distal renal tubular acidosis	34	0.4	32	2
3337	Fanconi syndrome, primary	19	0.2	14	5
91500	Tubulointerstitial nephritis and uveitis syndrome	16	0.2	12	4
31043	Familial primary hypomagnesemia with hypercalciuria and nephrocalcinosis	14	0.2	13	1
9999967*	Fanconi syndrome, induced by other drug	10	0.1	10	
Thrombotic Microangiopathies (OC 93573)		321	4.2	283	38
90038	Shiga toxin-associated hemolytic uremic syndrome	167	2.2	165	2
544472	Atypical hemolytic uremic syndrome with complement gene abnormality	57	0.7	46	11
9999987*	Atypical hemolytic uremic syndrome, not further specified	48	0.6	31	17
93581	Atypical hemolytic uremic syndrome with anti-factor H antibodies	19	0.2	18	1
544493	Streptcoccous pneumoniae-associated hemolytic uremic syndrome	11	0.1	11	
93585	Acquired thrombotic thrombocytopenic purpura	7	0.1	2	5
Metabolic Nephropathies (OC 93593)		302	3.9	233	69
411629	Infantile nephropathic cystinosis	84	1.1	67	17
93598	Primary hyperoxaluria type 1	53	0.7	50	3
324	Fabry disease	32	0.4	5	27
534	Lowe syndrome	24	0.3	23	1
93622	Dent disease type 1 (CLCN5-related)	22	0.3	17	5
300547	Autosomal recessive infantile hypercalcemia	18	0.2	16	2
27	Methylmalonic acidemia, Vitamin B12-unresponsive	18	0.2	16	2
411634	Juvenile nephropathic cystinosis	11	0.1	9	2
Rare Causes of Hypertension (OC 93618)		79	1.0	75	4
97598	Renal artery stenosis, congenital	41	0.5	38	
904	Williams syndrome	19	0.2	19	
636	Neurofibromatosis type 1	12	0.2	11	1

Percent values relate to total number of patients in registry (N = 7607)

In order to correctly capture complex diseases, in particular those with congenital anomalies of the kidneys and the urinary tract (CAKUT), assignment of up to two disease codes per patient is allowed. So far a second renal diagnosis has been coded in 481 subjects (6.5%), thereof 436 CAKUT patients. In 104 of the 1781 CAKUT patients (5.9%) the renal phenotype is part of a defined syndromic disorder.

### Patient population

The characteristics of the registry cohort collected during the first 2 years of the registry are summarized in Table 2. Structural kidney disorders (CAKUT and ciliopathies) and—acquired and hereditary—glomerulopathies represented the largest groups of rare kidney diseases regarding the numbers of patients and disease entities. Male gender was overrepresented in CAKUT, glomerulopathies (67% males in IgA nephropathy, 66% in membranous nephropathy, 62% in idiopathic steroid sensitive nephrotic syndrome), and metabolic nephropathies (because of male-exclusive Lowe syndrome).

**Table 2:** Characteristics of registry cohort

	CAKUT	Ciliopathies	Glomerulo-pathies	Tubulopathies	Metabolic nephropathies	Thrombotic micro-angiopathies	Rare causes of hypertension	All patients
No. of diagnoses (Orphacodes)	48	21	60	35	20	9	6	203
No. of patients (N (%))	1781 (24.1%)	1406 (19.0%)	2816 (38.1%)	680 (9.2%)	302 (4.1%)	321 (4.3%)	79 (1.1%)	7385 (100%)
Male sex (%)	68.5	47.3	56.5	52.4	57.6	51.1	49.3	57.0
Ethnicity (%)-Caucasian/Arabic/African/East Asian/other	86/8/3/1/2	93/4/1/1/1	90/5/2/2/1	79/9/2/1/9	81/13/1/2/3	90/4/3/0/3	86/9/0/0/5	87/6/2/1/4
Family history of kidney diseases (%)	5.3	46.1	11.4	21.7	16.9	3.4	11.4	16.8
Median (IQR) age at first signs or symptoms (years)	0.0 (0.0–0.2)	11.1 (0.6–28.0)	11.6 (3.8–41.0)	1.7 (0.2–9.2)	0.8 (0.1–2.9)	3.3 (1.3–6.9)	1.2 (0.3–4-4)	14.1(7.4–31.7)
Median (IQR) age at enrolment (years)	8.4 (3.3–13.9)	21.4 (11.4–46.8)	17.8 (10.7–50.9)	12.3 (6.6–18.7)	15.0 (7.8–24.5)	8.9 (4.7–14.9)	11.0 (6.4–14.9)	4.8 (0.3–18.5)
Genetic testing performed (% of all cases)	11.3	38	17.3	62.1	61.9	22.1	31.6	26.1
Genetic testing performed (% of hereditary disease cases)	21.2	38	60.7	65.2	61.9	72.3	58.5	46.3
Genetically confirmed diagnosis (% of all cases)	5.3	31.9	12.1	54.7	57.9	15.9	29.1	20.4
Genetically confirmed diagnosis (% of hereditary disease cases)	11.2	31.9	52.1	57.6	57.9	56.6	56.1	39.7
Genetically confirmed diagnosis (% of hereditary disease cases who underwent genetic screening)	52.0	84.1	85.5	88.2	93.6	78.3	95.8	85.8
Histopathologically confirmed diagnosis (%)	0.8	1.1	57.1	2.2	6.6	14.0	0	23.2
Disease stage (%) -CKD 1/2/3/4/5/Dialysis/Transplantation	36/23/13/5/2/3/17	36/21/15/7/7/4/10	52/19/11/4/1/3/9	68/21/8/1/1/1/1	41/22/14/5/1/5/13	43/22/11/5/2/4/13	80/14/4/0/0/0/3	47/20/12/5/2/3/11

Data are median (interquartile range) or N (%) as applicable

A family history for kidney disease was most commonly reported in ciliopathies (ADPKD), tubulopathies (nephrogenic diabetes insipidus) and metabolic nephropathies (X-linked hypophosphatemia). Genetic screening was performed in 26% of all patients and 46% of those with a hereditary disease diagnosis. A genetic disease cause was confirmed by testing in 20% of all patients and 40% of those with a hereditary disease. The mutation detection rate ranged from 96% in rare causes of hypertension and 94% in metabolic nephropathies to 52% in CAKUT disorders. Histopathological confirmation was obtained in 57% of glomerulopathies but was performed very rarely (0–2%) in CAKUT, ciliopathy and tubulopathy cases.

The disease groups distinctly differed with regards to global kidney function at time of enrolment: End-stage kidney disease was present in 22% of CAKUT, 21% of ciliopathy and 19% of thrombotic microangiopathy and metabolic nephropathy cases, as compared to 13% of glomerulopathies and 3% of patients with tubulopathies.

### Pediatric and adult disease characteristics

**Fig. 5:**
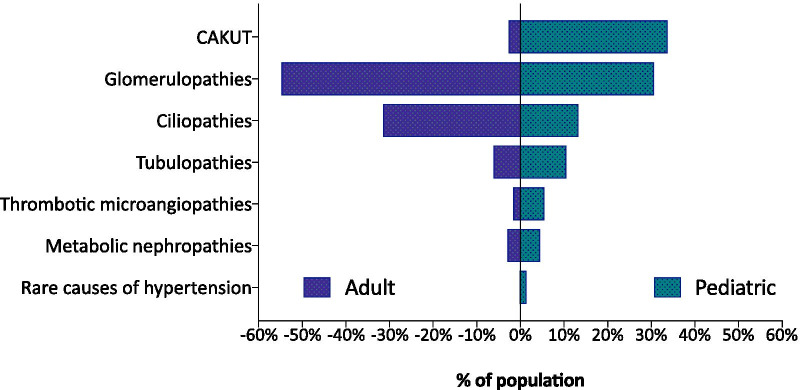
Distribution of disease groups in adult and pediatric patients

**Fig. 6:**
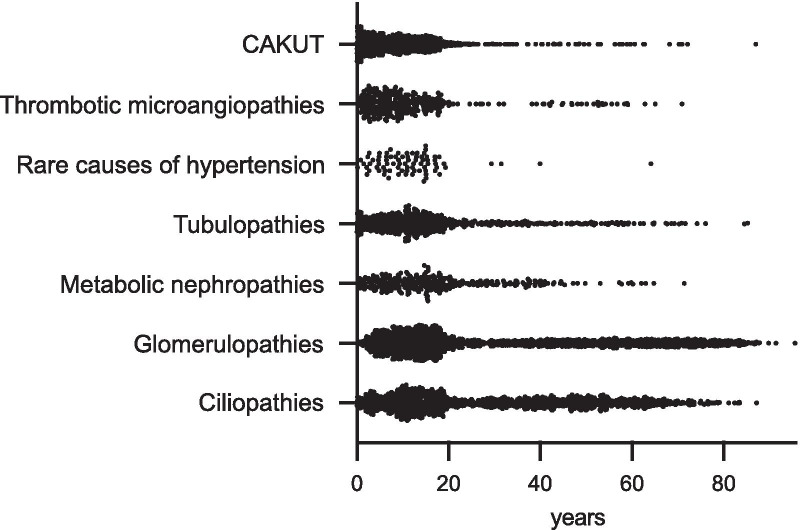
Distribution of age at time of enrolment per disease group

The distribution of rare kidney diseases differed markedly between pediatric and adult patients: whereas glomerulopathies (54.8%) and ciliopathies (31.5%, thereof 87.8% ADPKD) were the most common rare kidney diseases treated in adult nephrology units, the vast majority of CAKUT, tubulopathy, metabolic nephropathy and thrombotic microangiopathy patients were reported by pediatric units (Fig. 5). The distribution of patient age at time of enrolment is depicted in Fig. 6.

**Fig. 7:**
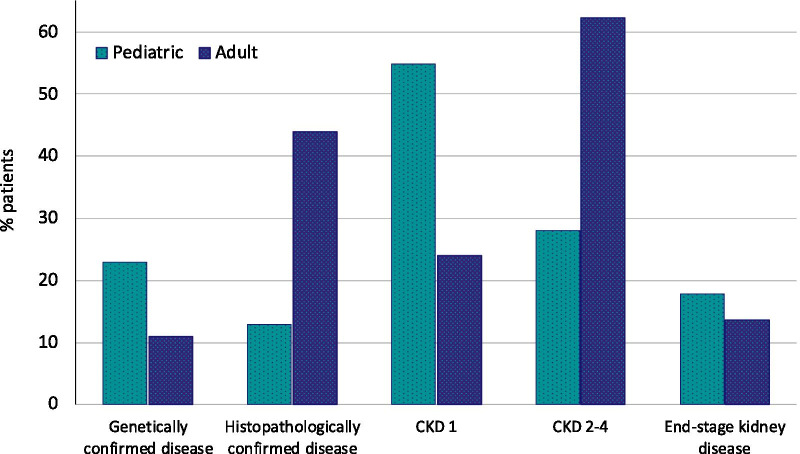
Characteristics of pediatric and adult patients. *CKD* chronic kidney disease stage

The proportion of genetic testing was 14% and 31% of all adult and pediatric patients, respectively. When considering only the genetic disease cases, the proportion of patients who underwent genetic testing was 28% in adults and 54% in pediatric patients. Pediatric patients had a genetically confirmed diagnosis twice as often as adult patients (24 vs 12%, Fig. 7). The most common genetically confirmed diagnoses were ADPKD, Alport syndrome, distal renal tubular acidosis, ARPKD and genetic steroid resistant nephrotic syndrome in pediatric patients and ADPKD, Alport syndrome, Fabry disease and Gitelman syndrome in adults.

In adults the renal diagnosis was secured by kidney biopsy in 44% of all cases as compared to 13% in pediatric patients. When considering only glomerulopathies, kidney biopsies were performed in 80.4% of the adult as compared to 38.5% of the pediatric cases. The most common histopathological diagnoses in adults were membranous nephropathy, IgA nephropathy, focal segmental glomerulosclerosis (FSGS), membranoproliferative glomerulonephritis (MPGN) and Lupus nephritis, and in pediatric patients, minimal change nephropathy, IgA nephropathy, FSGS and MPGN.

More than half of the pediatric patients had normal kidney function (54.8% CKD stage 1 vs 24% in adults) whereas the majority of adults were in CKD stage 2–4 (62% vs 28%). Similar fractions of patients had attained end-stage kidney disease at time of registry enrolment (pediatric: 17.8% vs adult: 13.6%).

## Discussion

ERKReg is among the first registries constructed by a European Reference Network to capture essential information from patients with all diagnoses in the thematic areas of interest. Since its launch in early 2019, the European Rare Kidney Disease Registry has rapidly grown to become the largest single database of patients with rare and complex kidney disorders assembled to date. With its well defined objectives, user friendly design and focused data collection, the registry has been well accepted and rapidly implemented by most consortium partners.

Some 300 rare disease entities with a renal phenotype are defined in the Orphanet classification system [10]. As expected, a highly asymmetric prevalence distribution of the individual kidney diseases was found, with 11 diagnoses covering 50% and 190 codes the other half of the population. Another 100 listed diseases are so rare that no cases were reported among the first 7600 patients. These findings reconfirm the need for large consortium of specialized healthcare services to join forces in order to collect sufficiently sized patient cohorts with rare and ultrarare kidney diseases for clinical research.

Based on patient counts performed when the ERKNet centres applied for membership, an estimated 25–30% of all available patients have been enrolled on average by the active centres to date, leaving room for further growth. Furthermore, the size of the ERKNet consortium is expected to nearly double in 2021 as some 30 centres with more than 30,000 patients have applied for ERKNet membership in the ongoing second call. All applicant sites have committed to enrolling their patients to ERKReg. Finally, the registry is also open to non-ERKNet sites, and a growing number of sites in non-EU countries have recently started contributing to the Registry. Hence, rapid further growth of the registry is expected.

The ERN registries are committed to the FAIR principles, i.e. aiming to make their databases **f**indable, **a**ccessible, **i**nteroperable, and **r**e-usable [12, 13]. The ERKReg registry website, metadata information including the registry’s data dictionary, and information about data access rules and procedures can easily be found by searching the Web. In addition, ERKReg is registered in the OrphaNet registry database and the European Rare Disease Registry Infrastructure (ERDRI) [14]. The metadata deployment of the registry’s data fields in the ERDRI.mdr repository is currently in progress. The integration of the Common Data Elements released by the EU’s Joint Research Centre (JRC) is an essential component of making the registry data interoperable [7] (Additional file 1: Table S1). An application programming interface (API) for automated processing of data queries through the emerging Virtual Platform of the European Joint Programme on Rare Diseases (EJP RD) is planned. In this way it is ensured to maximize the usefulness of the registry for the rare disease research community.

The presented summary of the baseline characteristics of the 7607 patients enrolled so far provides an estimate of the relative frequency of rare kidney disease groups and individual kidney diseases seen at specialized centres. In children, congenital anomalies of the kidney and urinary tract (CAKUT) and glomerulopathies represent the most common disease groups. In adult patients, glomerulopathies constitute the most common disease group, whereas ADPKD is the most common individual disease entity. The other main categories of rare kidney diseases, i.e. tubulopathies, metabolic nephropathies and thrombotic microangiopathies, altogether comprise only 21% of the patients seen in the pediatric and 11% of those in the adult nephrology units. This information, reflecting the relative clinical experience of rare kidney disease specialists with different types of disorders, is useful for the planning of educational but also clinical research activities by ERKNet and the rare kidney disease community at large.

**Fig. 8:**
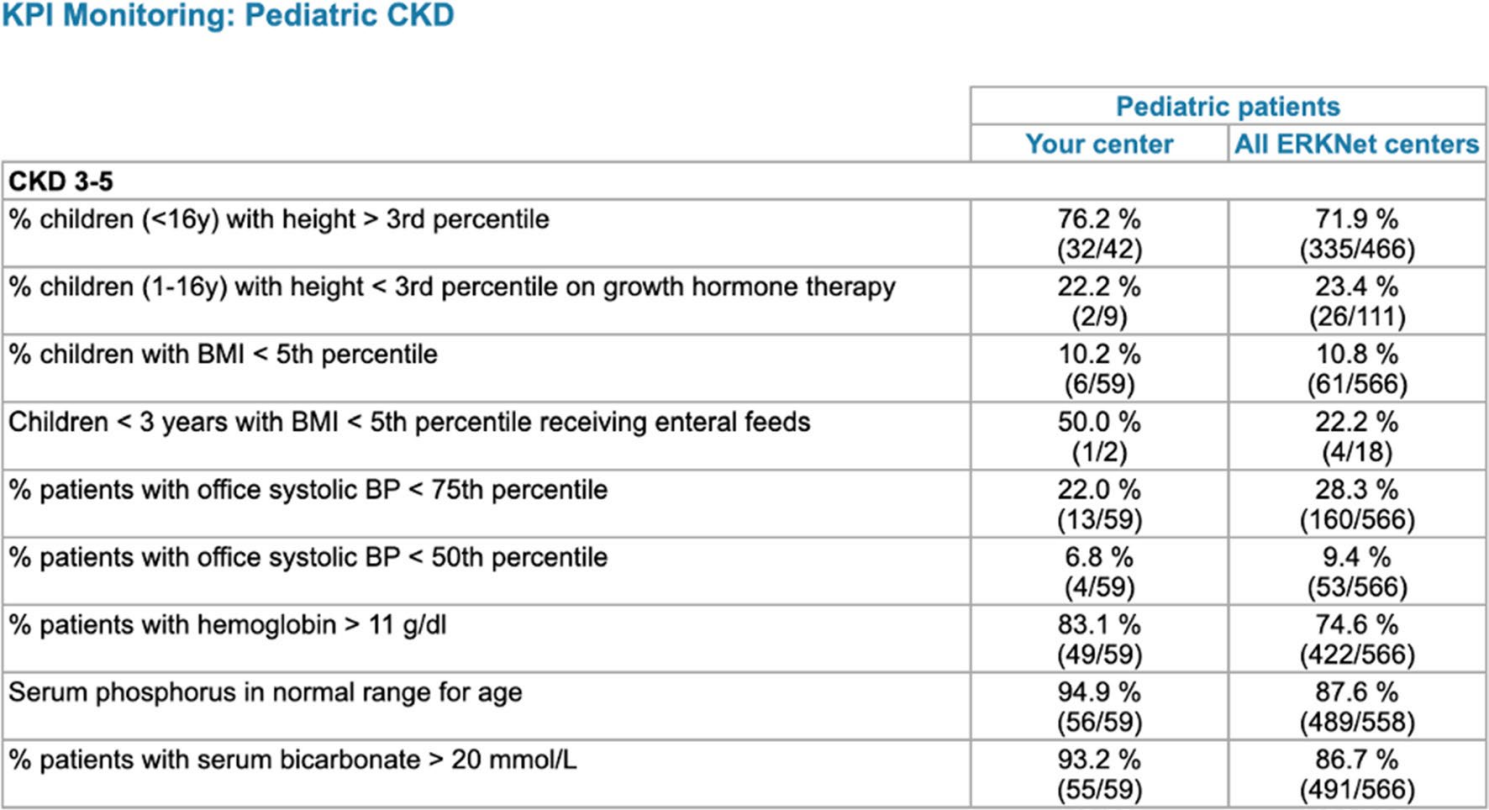
Example of Key Performance Indicator statistics and benchmarking feature as displayed on the Website to centre users

A unique feature of ERKReg is the KPI monitoring module. The selective, disease-specific capture of information concerning diagnostic and therapeutic management and outcomes is an efficient way to monitor clinical guideline adherence and patient outcomes. The KPIs were carefully selected by ERKNet experts in an iterative consensus process. The information of the clinicians regarding their centre’s performance in comparison with the registry averages (benchmarking, Fig. 8) is hoped to lead to gradual harmonization and optimization of patient management and improve patient outcomes across the Network. Concretely, such beneficial effects could encompass earlier genetic diagnostics to avoid ineffective and toxic immunosuppressive treatments, improved long-term preservation of kidney function by strict blood pressure control and pharmacological nephroprotection, minimized secondary morbidity by stringent compensation of tubular electrolyte and water losses, early treatment of bone-mineral disorder and renal anemia, and prevention of irreversible growth failure by growth promoting therapies in children.

ERKReg is designed as a core registry to identify patient cohorts for clinical research but does not include comprehensive data collection for individual disorders. However, thanks to its modular design ERKReg allows extended data collection in individual rare diseases of interest. For patients with a certain diagnosis enrolled in ERKReg, expanded eCRFs can optionally be selected. This concept has been applied successfully with a sub-registry for distal renal tubular acidosis and further disease-specific registries are under development. Hence, beyond its core purposes, ERKReg has the potential to become a platform for rare kidney disease registries.

The project has been facing several barriers and challenges (Table 3). Highly diverse feedback was received from the local Ethics Committees, ranging from no need for approval of a registry mainly serving healthcare optimization to requirements identical to a clinical trial including patient insurance despite no interventions being performed. The current fragmentation of ethics standards and evaluation processes across the EU member states and the local institutions is a major hurdle for EU-wide patient registries. While we eventually succeeded in meeting all individual demands, variable delays occurred that explain in part the rather heterogeneous national population coverage at this early stage of the registry’s operation as shown in Fig. 3. Furthermore, since ERKReg is expert centre- but not population-based, the registry cannot provide exact demographic figures about individual rare kidney diseases; this task will continue to require regional or national databases with compulsory/automated reporting by healthcare providers.

**Table 3:** Future objectives and challenges of the European Rare Kidney Disease Registry and disease-specific sub-registries

Objectives	Challenges
Epidemiology of rare kidney diseases Information on level of diagnostic ascertainment (inc. access to genetic testing) Phenotype and natural history information Continuous monitoring of diagnostic and therapeutic performance and guideline adherence for optimized patient outcomes Rapid identification of patient cohorts for clinical trials	Ensuring unbiased representation of the European rare kidney disease population Integration of EUPID pseudonymization system Link with electronic health records for automated data transmission Integration of quality of life data/disease-group specific patient reported outcome measures (PROMs) Long-term sustainability of registry

Another source of variation in the uptake of the registry were differences in available local expertise and available resources to organize the participation in an international registry. We tried to minimize local efforts by limiting data collection to the minimally required dataset, designing a user-friendly website and electronic case report form, training the local staff in videoconferences and by providing supportive material (such as a user guide and FAQ document) and establishing an online helpdesk. While these measures were largely successful, the registry still tended to be implemented more rapidly in pediatric than adult nephrology sites due to the pediatric centres’ frequently better staffing and greater familiarity with collaborative data collection. This resulted in a relative enrichment of pediatric over adult patients in the early phase of the registry. It is hoped that this imbalance will be largely compensated in the near future, in particular in view of the preponderance of adult nephrology sites applying in the ongoing second ERN call. Also, some ascertainment bias may have been caused by preferential enrolment of patients in rare kidney disease outpatient clinics with relatively short disease history and preserved kidney function. We hope that as the registry matures, patients with long-standing disease followed in dialysis units and transplant clinics will be progressively included as well.

Despite our efforts to minimize the data entry workload, the registry currently requires manual data submission. Automated data transmission is not (yet) possible due to the highly variable data structure and completeness at the centre level and the fragmentation of hospital IT systems. Pilot efforts to represent the JRC Common Data Elements in the electronic health records are currently starting in individual institutions. It is hoped that these activities will facilitate at least partially automated data capture in the foreseeable future.

The development of ERKReg parallels the efforts to establish registries in all 24 ERNs. The anticipated interoperability of the ERN registries will allow to search for patient groups with overlapping phenotypes that may be respresented in different ERN databases via the virtual platform developed by the EJP-RD consortium.

In conclusion, ERKNet has successfully created and implemented the European Rare Kidney Disease Registry as a core database comprising a representative sample of patients treated for rare kidney diseases in highly specialized pediatric and adult nephrology units throughout Europe. The availability of a single database comprising currently treated patients with more than 200 individual kidney diseases is hoped to expedite the identification of patient cohorts available for diagnostic, prognostic and therapeutic research.

## Supplementary Information

**Additional file 1: Table S1.** Representation of JRC common data elements in ERKReg.

**Additional file 2: Table S2.** List of key performance and outcome indicators (KPIs) in ERKReg Registry

## Data Availability

Up-to-date registry data is available online in the Annual Registry Reports (www.erkreg-reports.erknet.org). Further anonymized patient level data can be made available upon request. The data access request procedure and a data request form are available on the registry website.

## References

[1] Devuyst O, Knoers NV, Remuzzi G, Schaefer F (2014). Rare inherited kidney diseases: challenges, opportunities, and perspectives. Lancet.

[2] Wühl E, van Stralen KJ, Wanner C, Ariceta G, Heaf JG, Bjerre AK (2014). Renal replacement therapy for rare diseases affecting the kidney: an analysis of the ERA–EDTA Registry. Nephrol Dial Transplant.

[3] Héon-Klin V (2017). European Reference networks for rare diseases: what is the conceptual framework?. Orphanet J Rare Dis.

[4] European Rare Kidney Disease Reference Network (ERKNet). 2017. https://erknet.org/index.php?id=home. Accessed 27 Jan 2021.

[5] Kodra Y, Weinbach J, Posada-de-la-Paz M, Coi A, Lemonnier SL, Van Enckevort D (2018). Recommendations for improving the quality of rare disease registries. Int J Environ Res Public Health.

[6] Orphanet Report Series. Rare Diseases collection, September 2020. Rare Disease Registries in Europe. 2020. https://www.orpha.net/orphacom/cahiers/docs/GB/Registries.pdf. Accessed 27 Jan 2021.

[7] European Commission, EU RD Platform. Set of Common Data Element for Rare Diseases Registration. 2021. https://eu-rd-platform.jrc.ec.europa.eu/set-of-common-data-elements_en. Accessed 28 Jan 2021.

[8] European Medicines Agency. Note for guidance on good clinical practice (CPMP/ICH/135/95). 2002. https://www.ema.europa.eu/en/ich-e6-r2-good-clinical-practice. Accessed 27 Jan 2021.

[9] European Union. Regulation (EU) 2016/679 of the European Parliament and of the Council of 27 April 2016 on the protection of natural persons with regard to the processing of personal data and on the free movement of such data, and repealing Directive 95/46/EC (General Data Protection Regulation). 2016. https://eur-lex.europa.eu/eli/reg/2016/679/oj. Accessed 27 Jan 2021.

[10] Orphanet Report Series, Rare Diseases collection, January 2021. List of rare diseases and synonyms listed in alphabetical order. 2021. https://www.orpha.net/orphacom/cahiers/docs/GB/List_of_rare_diseases_in_alphabetical_order.pdf. Accessed 27 Jan 2021.

[11] The European Rare Kidney Disease Registry (ERKReg). Orphacode renal diagnosis catalogue. 2021. https://erknet.org/index.php?id=282. Accessed 27 Jan 2021.

[12] Blumenthal S (2018). Improving Interoperability between Registries and EHRs. AMIA Jt Summits Transl Sci Proc.

[13] Schaaf J, Kadioglu D, Goebel J, Behrendt CA, Roos M, van Enckevort D (2018). OSSE Goes FAIR—Implementation of the FAIR Data Principles for an Open-Source Registry for Rare Diseases. Stud Health Technol Inform.

[14] European Commission, EU RD Platform. European Rare Disease Registration Infrasctructure (ERDRI) 2020. https://eu-rd-platform.jrc.ec.europa.eu/erdri-description_en. Accessed 27 Jan 2021.

