# Supplementary material for: The European Rare Kidney Disease Registry (ERKReg): objectives, design and initial results
Source: Orphanet J Rare Dis. 2021 Jun 2;16:251. doi: 10.1186/s13023-021-01872-8 (PMC8173879; doi:10.1186/s13023-021-01872-8)
Supplement: Supplementary file 1 — Additional file 1: Table S1. Representation of JRC common data elements in ERKReg. [file 13023_2021_1872_MOESM1_ESM.docx]

**Supplementary Table 1.** Representation of JRC common data elements in ERKReg.

| **Group** | **Element** | **Element Name** | **Field name in ERKReg** | **ERKReg coding** |
| --- | --- | --- | --- | --- |
| 1  Pseudonym | 1.1 | Pseudonym | pat_id | string |
| 2  Personal Information | 2.1 | Date of birth | birthdate | yyyy/mm |
|  | 2.2 | Sex | sex | M=Male, F=Female |
| 3  Patient´s status | 3.1 | Date of status change | cr_term_date | dd/mm/yyyy |
|  |  | Status type (if not currently followed) | term_reason | blank= alive and followed in registry  1=loss of follow-up  2=patient death  3=Transition to adult unit  4=Transition to other ERKReg center |
|  | 3.2 | Date of death | death_date | dd/mm/yyyy |
| 4  Care Pathway | 4.1 | First contact with specialized center | first_visit_date | dd/mm/yyyy |
| 5  Disease History | 5.1 | Age of onset | first_symptoms_date | yyyy/mm |
|  | 5.2 | Age at diagnosis | dia_date | dd/mm/yyyy |
| 6  Diagnosis | 6.1 | Diagnosis of the rare disease | diagnosis  diagnosis2nd | Orpha Code  Orpha Code |
|  | 6.2 | Genetic diagnosis | gen_diagnosis | 0= No, 1= Yes, 9= Result pending  HGVS classification system |
|  | 6.3 | Undiagnosed case | dia_est  if no: signs and symptoms compatible with symptoms_group | 0= No, 1= Yes  Orpha Code |
| 7  Research | 7.1 | Agreement to be contacted for research purposes | informed_consent_date  consent_research | dd/mm/yyyy  0=No, 1=Yes |
|  | 7.2 | Consent to the reuse of data | informed_consent_date  consent_registry | dd/mm/yyyy  0=No, 1=Yes |
|  | 7.3 | Biological sample | Not implemented in ERKReg | |
|  | 7.4 | Link to a databank | Not implemented in ERKReg | |
| 8  Disability status | 8.1 | Disability score | Not implemented in ERKReg | |
