# Supplementary material for: The European Rare Kidney Disease Registry (ERKReg): objectives, design and initial results
Source: Orphanet J Rare Dis. 2021 Jun 2;16:251. doi: 10.1186/s13023-021-01872-8 (PMC8173879; doi:10.1186/s13023-021-01872-8)
Supplement: Supplementary file 2 — Additional file 2: Table S2. List of key performance and outcome indicators (KPIs) in ERKReg Registry [file 13023_2021_1872_MOESM2_ESM.docx]

**Supplementary Table 2.** List of key performance and outcome indicators (KPIs) in ERKReg Registry

| General |
| --- |
| Time (months) from 1^st^ symptom to diagnosis: Median (IQR) |
| Time (months) from referral to diagnosis: Median (IQR) |
| % hereditary disease patients with any genetic screening |
| % hereditary disease patients with NGS screening (panel, WES) |

| Glomerulopathies |
| --- |
| % patients with hereditary glomerulopathies with genetic screening |
| % patients with multidrug resistant NS with comprehensive genetic screening |
| % normotensive patients (CKD1-3) |
| % patients with office blood pressure in target range |
| % patients with immunological glomerulopathies in clinical remission |
| % patients with persistent proteinuria who receive RAS antagonist therapy |
| % patients with hereditary and multidrug resistant NS on conservative treatment who are prescribed RAS antagonist therapy |
| % adult patients with statin therapy |
| % adult patients with LDL cholesterol <100 mg/dl |
| % children with steroid sensitive idiopathic nephrotic syndrome who are obese |
| % children with steroid sensitive idiopathic nephrotic syndrome with height < 3rd percentile |

| Tubulopathies & metabolic nephropathies |
| --- |
| % patients with hereditary nephropathy with genetic confirmation |
| % children (<16y) with normal length/height SDS |
| % patients with renal tubular acidosis maintaining normal serum bicarbonate |
| % cystinosis patients in whom at least one cystine blood level has been obtained during the past 12 months |
| % cystinosis patients with leukocyte cystine level < 1 nmol/mg protein |
| % cystinuria patients free of new stones in past 12 months |
| % Fabry disease patients with at least one proteinuria measurement in past 12 months |
| % Bartter/Gitelman patients with serum K > 2.5 mmol/l |

| Thrombotic microangiopathies |
| --- |
| % new-onset HUS patients with complete initial diagnostics pediatric: ADAMTS13, STEC stool culture, Shigatoxin PCR adult: ADAMTS13 only |
| % aHUS patients with genetic/autoantibody screening (NGS, CFH autoantibodies) |

| Structural kidney disorders |
| --- |
| % ADPKD patients with genetic screening |
| % ADPKD patients with at least one total kidney volume measurement by MRI |
| % normotensive ADPKD patients (CKD1-4) by office BP |
| % ADPKD patients screened for intracranial aneurysm when family history is positive |
| % TSC patients treated with mTOR inhibitors for indication AML>3 cm |
| % TSC patients treated with mTOR inhibitors for neurological indication (SEGA/Epilepsy) |
| % VHL patients with children or planning a pregnancy offered prenatal genetic counseling |
| % VHL patients with regular audiology assessment (every 2 years, starting at age 5) |

| CAKUT, ciliopathies & obstructive uropathies |
| --- |
| % familial cystic disease patients with genetic screening |
| % patients with PUV detected by prenatal ultrasound |
| Mean (SD) of febrile UTIs in past 12 months in OUP / VUR patients |

| Pediatric CKD 3-5 |
| --- |
| % children (<16y) with height > 3rd percentile |
| % children (1-16y) with height < 3rd percentile on growth hormone therapy |
| % children with BMI < 5th percentile |
| % Children < 3 years with BMI < 5th percentile receiving enteral feeds |
| % patients with office systolic BP < 75th percentile |
| % patients with office systolic BP < 50th percentile |
| % patients with hemoglobin > 11 g/dl |
| Serum phosphorus in normal range for age |
| % patients with serum bicarbonate > 20 mmol/L |

| Pediatric dialysis |
| --- |
| % children (<16y) with height > 3rd percentile |
| % children (1-16y) with height < 3rd percentile on growth hormone therapy |
| % children with BMI < 5th percentile |
| Children < 3 years with BMI < 5th percentile receiving enteral feeds |
| Serum phosphorus in normal range for age |
| % patients with hemoglobin > 11 g/dl |
| % patients with serum bicarbonate > 20 mmol/L |
| PD peritonitis rate |

| Pediatric transplantation |
| --- |
| % children with pre-emptive transplantation |
| % children (<16y) with height > 3rd percentile |
| % children (1-16y) with height < 3rd percentile on growth hormone therapy |
| % normotensive children (by office BP) |
| % patients with hemoglobin > 10 g/dl |
| % patients without severe metabolic acidosis |
| % patients wih biopsy proven rejection |
